# Expression of myriapod pair rule gene orthologs

**DOI:** 10.1186/2041-9139-2-5

**Published:** 2011-02-25

**Authors:** Ralf Janssen, Graham E Budd, Nikola-Michael Prpic, Wim GM Damen

**Affiliations:** 1 Uppsala University, Department of Earth Sciences, Palaeobiology, Villavägen 16, SE-752 36 Uppsala, Sweden; 2 Georg-August-Universität Göttingen, Johann-Friedrich-Blumenbach Institut für Zoologie und Anthropologie, Abteilung für Entwicklungsbiologie, GZMB Ernst-Caspari-Haus, Justus-von-Liebig-Weg 11, 37077 Göttingen, Germany; 3 Friedrich-Schiller-University Jena, Department of Genetics, Philosophenweg 12, D-07743 Jena, Germany

## Abstract

**Background:**

Segmentation is a hallmark of the arthropods; most knowledge about the molecular basis of arthropod segmentation comes from work on the fly *Drosophila melanogaster*. In this species a hierarchic cascade of segmentation genes subdivides the blastoderm stepwise into single segment wide regions. However, segmentation in the fly is a derived feature since all segments form virtually simultaneously. Conversely, in the vast majority of arthropods the posterior segments form one at a time from a posterior pre-segmental zone. The pair rule genes (PRGs) comprise an important level of the *Drosophila *segmentation gene cascade and are indeed the first genes that are expressed in typical transverse stripes in the early embryo. Information on expression and function of PRGs outside the insects, however, is scarce.

**Results:**

Here we present the expression of the pair rule gene orthologs in the pill millipede *Glomeris marginata *(Myriapoda: Diplopoda). We find evidence that these genes are involved in segmentation and that components of the hierarchic interaction of the gene network as found in insects may be conserved. We further provide evidence that segments are formed in a single-segment periodicity rather than in pairs of two like in another myriapod, the centipede *Strigamia maritima*. Finally we show that decoupling of dorsal and ventral segmentation in *Glomeris *appears already at the level of the PRGs.

**Conclusions:**

Although the pair rule gene network is partially conserved among insects and myriapods, some aspects of PRG interaction are, as suggested by expression pattern analysis, convergent, even within the Myriapoda. Conserved expression patterns of PRGs in insects and myriapods, however, may represent ancestral features involved in segmenting the arthropod ancestor.

## Background

The expression of the pair rule genes (PRGs) in seven transversal stripes is the first sign of metamerization in *Drosophila*. Primary PRGs are regulated in a double segmental periodicity by upstream-acting maternal-effect genes and zygotically-expressed gap genes at the blastoderm stage. The primary PRGs then regulate the expression of the secondary PRGs in a similar double segmental pattern (reviewed in [1,2]). This expression pattern in alternating segments is also reflected in the phenotypes of null mutants of these genes: the loss of alternating segmental structures (hence, the name "pair rule") [3]. Later in the extended germ band stage secondary stripes of many PRGs intercalate between the primary stripes and the genes function as segment polarity genes at this time of development [4,5].

However, this mode of segmentation where all segments are produced simultaneously (the so-called long-germ developmental mode) is derived within the insects and is apparently correlated with the high speed of *Drosophila *embryonic development [6,7]. In the majority of arthropods (including many holometabolous insects) only the anterior segments form simultaneously, and all posterior segments are formed sequentially from a posterior segment addition zone (SAZ) [8]. This mode of segment formation is called the short-germ developmental mode. Theoretically, this mode of sequential segment formation does not require the pair rule gene mechanisms operating during *Drosophila *segmentation. However, studies in the beetle *Tribolium castaneum *have shown that although the developmental mode is that of a typical short-germ arthropod, some of the PRG orthologs function as "true" pair rule genes [9,10]. Functional studies in more basal hemimetabolous insects, such as *Oncopeltus fasciatus *or *Gryllus bimaculatus*, support the idea that PRGs are involved in segmentation, but question the existence of a pair rule mechanism [11,12]. Studies on the expression of PRGs in chelicerates [13], crustaceans [14,15] and myriapods [16] support this thesis. An exception is seen in the centipede *Strigamia maritima*, where posterior segments are initially determined in a two-segmental periodicity, revitalizing the question of whether an ancestral pair rule mechanism might exist in arthropods [17,18].

Part of the hierarchic interactions of the PRGs as known from *Drosophila *is also conserved in the beetle *Tribolium *[9,10] and may also be conserved in a spider [13]. However, some levels of PRG interaction are obviously divergent and it is, thus, unclear to what extent the interactions of PRGs are generally conserved in arthropods.

In this paper we present the expression profiles of orthologs of most of the known *Drosophila *PRG orthologs in the pill millipede *Glomeris marginata *during trunk segmentation. The data support the idea that PRG orthologs are generally involved in segmentation, but do not function as classical pair rule genes like in *Drosophila *and *Tribolium*. Our data show that posterior segments are formed one at a time and not in a double segmental periodicity. The interaction of PRG orthologs in *Glomeris*, as inferred from gene expression data, displays more similarities to *Tribolium *than to *Drosophila*. Also some of the PRG orthologs appear to have either ventral or dorsal specific functions, suggesting that the earlier reported decoupling of ventral and dorsal segmentation [19,20] is already obvious with the function of the PRGs. Finally, we discuss our data in the context of the assumed arthropod segmentation clock acting in the addition of posterior segments under the control of Delta-Notch signaling.

## Materials and methods

### Gene cloning

RNA isolation and cDNA synthesis were carried out as described previously [19]. Initial fragments of the *Glomeris even-skipped, runt and hairy-1 *gene orthologs were amplified using degenerate primer sets directed against the homeodomain (for *even-skipped*), the runt domain (for *runt*) and the helix-loop-helix domain (for *hairy*), respectively, as described by Damen *et al. *2000 [21]. The degenerate primers for the isolation of *Glomeris sloppy-paired*, *odd-paired*, *odd-skipped *and *pairberry-1 *orthologs have been described in Damen *et al. *2005 [13] and Schoppmeier and Damen 2005 [22]. To obtain larger fragments of the genes we subsequently performed 3'-RACE PCR using the MARATHON RACE Kit (Clontech, Heidelberg, Germany).

Sequences of all fragments were determined from both strands of several clones on a 3100 automated sequencer, using Big Dye dye-terminators version 3.1 (Big Dye Terminator Cycle Sequencing Kit; Perkin-Elmer Applied Biosystems, Foster City, CA, USA). The sequences of *Glomeris *pair rule gene orthologs are available under the accession numbers AM279687 (*Gm-eve*), AM279688 (*Gm-h1*), FR715033 (*Gm-h2*), FR715033 (*Gm-h3*), AM279692 (*Gm-run*), FR715039 (*Gm-slp*), FR715035 (*Gm-opa*), FR715036 (*Gm-odd*), FR715037 (*Gm-pby1*) and FR715038 (*Gm-pby2*).

### Sequence analysis

For the similarity analysis, we searched GenBank [23] using the pairwise alignment program Gapped BLAST [24]. Sequences were aligned using the multiple alignment program Clustal X [25]. The alignments were calculated from the blocks substitution matrix BLOSUM 62 using gap costs at 20 for existence and 0.2 for extension. Maximum likelihood analysis was performed using the Quartet Puzzling method [26] as implemented in PAUP 4.0b10 [27].

### In situ hybridization and nuclei staining

Whole mount *in situ *hybridizations were performed as described in Prpic and Tautz (2003) [28]. Double *in situ *staining with digoxigenin (DIG) and fluorescein (FL) labeled RNA probes in parallel is described in Janssen *et al. *(2008) [29]. For reasons of enhanced signal clearness (yolk stains yellow when using INT/BCIP or FastRed) embryos were flat mounted prior to photography. A detailed *in situ *hybridization protocol is available from the authors upon request.

Cell nuclei distribution was visualized by using the fluorescent dye 4-6-Diamidin-2-phenylindol (DAPI). Incubation in 1 μg/ml DAPI in phosphate buffered saline with 0.1% Tween-20 (PBST) for 30 minutes was followed by extensive washes in PBST. Embryos were analyzed under a Leica dissection microscope (Leica, Heerbrugg, Switzerland) equipped with either an Axiocam (Zeiss, Jena, Germany) or a D70 digital camera (Nikon, Tokyo, Japan). Brightness, contrast, and color values were adjusted in all images using the image processing software Adobe Photoshop CS2 (Version 9.0.1 for Apple Macintosh (Adobe Systems Inc. San Jose, CA, USA).

## Results

### Transcripts and phylogenetic analysis

**Figure 1:**
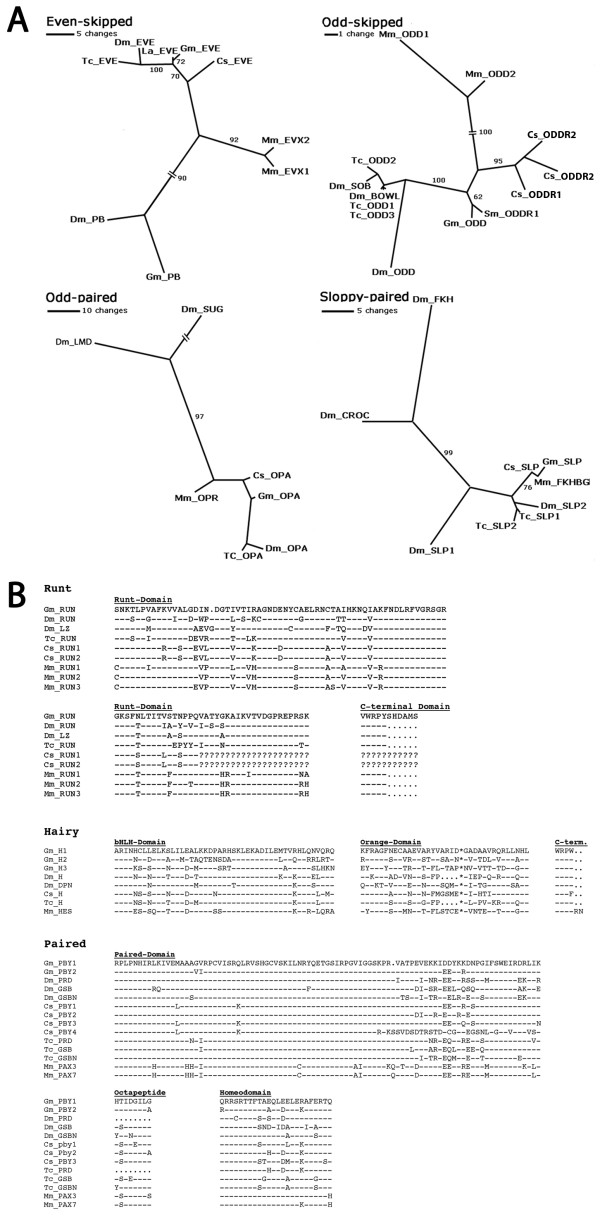
**Phylogenetic analysis of *Glomeris *pair rule gene orthologs**. **(A) **Phylograms of orthologs of the *Drosophila *pair rule genes Even-skipped, Odd-skipped, Odd-paired and Sloppy-paired from various arthropods and the mouse. Closely-related genes from the *Drosophila *genome serve as outgroups. Un-rooted majority rule consensus trees are shown, computed from 1000 intermediate trees produced with the Quartet Puzzling Method [25]. Reliability values are indicated at the edges. Abbreviations for animals: *Cs, Cupiennius salei *(spider); *Dm, Drosophila melanogaster *(fly); *Gm, Glomeris marginata *(millipede*); La, Lithobius atkinsoni *(centipede); *Mm, Mus musculus *(mouse); *Sm; Strigamia maritima *(centipede); *Tribolium castaneum *(beetle). Abbreviations for gene names: LMD, Lame duck; SUG, Sugarbabe; BOWL, Brother of odd with entrails limited; CROC, Crocodile; EVE, Even-skipped; EVX, vertebrate Even-skipped ortholog; FKH, Forkhead; FKHBG, Forkhead box G1; ODD, Odd-skipped; ODDR, Odd-skipped-related; OPA, Odd-paired; OPR, Odd-paired-related; PB, Proboscipedia; SLP, Sloppy-paired; SOB, Sister of odd and bowl. (**B) **Alignment of Runt, Hairy and Paired sequences. Orthologs from *Drosophila melanogaster*, *Tribolium castaneum*, *Cupiennius salei *and mouse are aligned with the available *Glomeris marginata *gene fragments. Aligned is part of the Runt domain and the C-terminal consensus (for Runt); part of the bHLH domain and the orange domain (for Hairy); part of the Paired domain, the octapeptide and part of the homeodomain (for Paired/Pairberry). Dashes denote identical amino acids, dots indicate gaps introduced to improve the alignment. Question marks stand for missing sequence information. Species abbreviations as in (A). Gene name abbreviations as in (A). Additional abbreviations: DPN, Deadpan; GSB, Gooseberry; GSBN, Gooseberry-neuro; Pax3/7, Pax group III genes; PRD, Paired; H, Hairy; HES, Hairy/Enhancer of split; LZ, Lozenge; PBY, Pairberry; RUN, Runt.

We recovered gene fragments with significant similarity to the *even-skipped*, *runt*, *sloppy-paired*, *odd-skipped *and *odd-paired *genes from *Drosophila melanogaster *and other arthropods that we designated as *Glomeris even-skipped *(*Gm-eve*), *Glomeris runt *(*Gm-run*), *Glomeris sloppy-paired *(*Gm-slp*), *Glomeris odd-skipped *(*Gm-odd*) and *Glomeris odd-paired *(*Gm-opa*), respectively (Figure 1). The *Gm-eve *fragment covers almost the complete homeodomain (except its very N-terminal end), the complete C-terminal part of the open reading frame (ORF) and a subsequent 3'untranslated region (UTR) ending in a poly-A tail. Within a reasonable distance from the poly-A sequence is a putative polyadenylation signal (AATAAA). The *Gm-run *fragment covers most of the runt-domain (except its N-terminal end), the complete C-terminal part of the ORF including the characteristic C-terminal WRPY motif (Figure 1B) [30]. Remarkably, this C-terminal WRPY motif is not at the very end of the ORF in *Glomeris *as it is in other arthropod and vertebrate *runt *orthologs, but is followed by a sequence of an additional six amino acids (Figure 1B). The subsequent 3'UTR ends in a poly-A stretch, but an obvious polyadenylation signal is not present.

The *Gm-slp *fragment encodes the C-terminal part of the protein including part of the forkhead-domain and the complete 3'-UTR as indicated by the presence of a putative polyadenylation signal (AATAAA). *Gm-odd *and *Gm-opa *encode part of the zinc-finger domains. The 5'and 3'ends of these two genes have not been recovered.

We isolated three gene fragments that show significant similarities to *Drosophila hairy *(Figure 1B). *Drosophila *possesses several bHLH transcription factors related to *hairy *with *deadpan *and *side *showing most similarities to *hairy *(Figure 1B). Since the order of branches in our phylogenetic analysis is statistically unresolved (trees not shown; cf. [21]) we designated the *Glomeris *fragments simply as *Gm-h1*, *Gm-h2*, and *Gm-h3*. All fragments encode the bHLH-domain (except its basic N-terminal part), the orange-domain, and the complete C-terminal part of the ORF each ending in a WRPW motif which, like the orange-domain, is characteristic for the *hairy*-class of bHLH-domain genes. All *Glomeris hairy *fragments contain a 3'UTR ending in a poly-A tail with a putative polyadenylation signal (that is, AATAAA for *Gm-h1 *and ATTAAA for *Gm-h2 *and *Gm-h3*) just upstream.

We obtained partial fragments for two *Glomeris *PaxIII group genes, which we named *pairberry-1 *(*Gm-pby1*) and *pairberry-2 *(*Gm-pby-2*) because of their sequence similarity to the *Drosophila *PaxIII group genes (that is, *paired*, *gooseberry-neuro *and *gooseberry*). The conceptually translated protein fragment contains the paired-domain (except its very N-terminal part), the octapeptide domain and the homeodomain (except its very C-terminal part) (Figure 1B).

### Expression of primary PRG orthologs during Glomeris trunk segmentation

Three of the known pair rule genes (PRGs) from *Drosophila*, that is, *even-skipped*, *runt *and *hairy*, are described as primary since they act upstream of the so-called secondary PRGs and control the expression of the latter [1]. The following section focuses on the expression of the orthologs of the primary PRGs during trunk segmentation in *Glomeris*.

**Figure 2:**
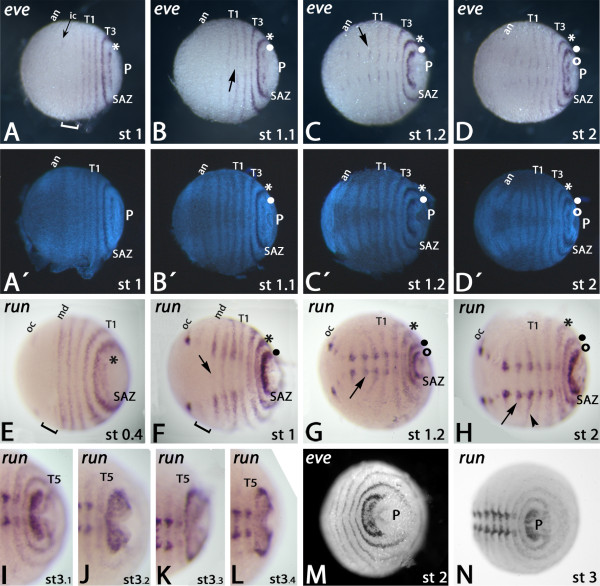
**Expression of *Glomeris even-skipped *and *runt***. Expression of *even-skipped *(**A-D, M**) and *runt *(**E-L, N**). (**A'-D') **show DAPI counter-staining of the embryos shown in (A-D). (**A) **Stage 1 embryo. Segmental stripes in every segment. Bracket marks premandibular and mandibular segments. Asterisk marks stripe of expression in the SAZ. (**B) **Stage 1.1 embryo. Arrow points to fading ventral expression. Note that the expression in the SAZ in (A) (asterisk) is now in the nascent segment anterior to the SAZ (asterisk in (B)) and a new expression domain is forming in the SAZ (filled circle). (**C) **Stage 1.2 embryo. Arrow points to expression in the CNS. Note that the expression domain seen in the SAZ in (B) has developed into a single broad domain (filled circle). (**D) **Stage 2 embryo. Former expression in the SAZ has transformed into a stripe in the nascent segment (filled circle). A new expression domain forms in the SAZ (open circle). (A'-D') DAPI counter-staining of the embryos shown in (A-D). **(E) **Stage 0.4 embryo. Bracket marks premandibular and antennal segments. Expression in the SAZ (asterisk). (**F) **Stage 1 embryo. Arrow points to disappearing ventral expression. Expression seen in the SAZ in A now lies in the nascent segment anterior to the SAZ (asterisk). A new domain of expression has formed in the SAZ (filled circle). Bracket as in (E). (**G) **Stage 1.2 embryo. Arrow points to expression in the CNS. Expression in the SAZ seen in (F) is now in the nascent segment anterior to the SAZ (filled circle). Expression seen in the SAZ in (E) (asterisk) is now one segment anterior to it. A new domain appears in the SAZ (open circle). (**H) **Stage 2 embryo. Arrow as in (G). Expression in the SAZ (open circle) expands. Arrowhead points to strengthened expression in the postmaxillary segment. (**I-L**) Dynamic expression of *runt *in the SAZ in four consecutive developmental stages (that is, stage 3.1 to 3.4). (**M, N) **Concentric expression of *even-skipped *(M) and *runt *(N) around the proctodaeum. In all pictures anterior is to the left. Abbreviations: an, antennae; ic, intercalary (premandibular) segment; md, mandibular segment; oc, ocular field; P, proctodaeum; SAZ, segment addition zone; T, trunk segment.

**Figure 3:**
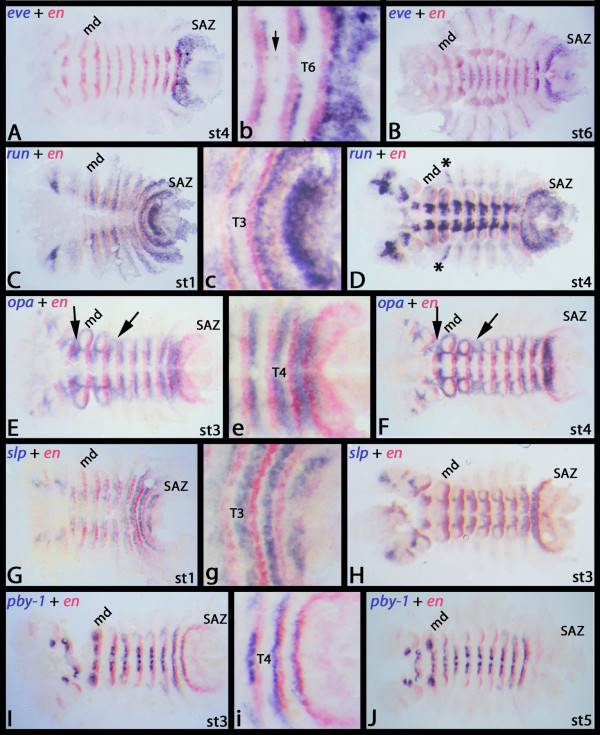
**Double staining of *Glomeris *PRGs (purple) with *engrailed *(red)**. Pictures denoted in capital letters are whole mounts; pictures denoted in small letters show close-ups. All embryos are flat-mounted; yolk removed. (**A, B) ***engrailed *(*en*) is expressed anterior adjacent to *even-skipped *(*eve*). Arrow in (B) points to dot-like expression in the CNS. (**C, D) ***en *is expressed anterior adjacent to *runt (run)*. Asterisks in (D) denote enhanced dorsal expression in the postmaxillary segment. (**E, F) ***en *is expressed posteriorly adjacent to *opa*. The arrows mark *opa *expression at the position where the premandibular and postmaxillary appendages should form, but are repressed by unknown mechanisms. (**G, H) ***en *is expressed posterior to *sloppy-paired *(*slp*) at the anterior edge of the SAZ. (**I, J) ***en *is expressed posterior to and partially overlapping with *pairberry-1 *(*pby-1*) in nascent segments. Abbreviations: md, mandibular segment; SAZ, segment addition zone; st, stage; T, trunk segment.

*Gm-eve *is expressed in segmental stripes. During the process of segment formation it is dynamically expressed in the SAZ (Figures 2 A-D, M and 3A, B). This dynamic expression starts with two small patch-like expression domains on either side of the proctodaeum in the posterior part of the SAZ (Figure 2B, D) that soon after broadens into a single patch-like expression domain (Figure 2A) and finally refines into a single stripe of expression in the very anterior of the nascent segment and posterior adjacent to the segmental expression of *engrailed *(*en*) (Figures 2A-D, M and 3A, B). This segmental *eve *expression covers the ventral and dorsal tissue in nascent segments and the SAZ (Figure 2C, D). The dorsal segmental expression persists slightly longer than the ventral (Figure 2C, D). *eve *is expressed in circles surrounding the proctodaeum in the most posterior segments (Figures 2M and 3A). At later stages this is less obvious since the SAZ and its surrounding tissue narrows. Apart from its expression during segment formation, *Gm-eve *is also expressed in the developing ventral nervous system (CNS) (Figure 2C, D).

*Gm-run *is expressed in segmental stripes at early segmentation stages (Figure 2E). Like *Gm-eve*, *Gm-run *is also dynamically expressed in the SAZ (Figures 2E-L, N and 3C, D). In contrast to the dynamic expression of *eve*, *run *initially appears in a small domain in the SAZ anterior adjacent to the proctodaeum (Figures 2E, G and 3C), that later broadens and finally fills the complete SAZ (Figure 2F, H). Then this domain disappears from the SAZ and only leaves a distinct stripe of expression in the very anterior of the nascent segment (Figures. 2K and 3C, D). The ventral portion of this stripe disappears soon after while the dorsal part persists (Figure 2L). As described for *eve*, the expression of *run *in the most posterior segments is also in circles (Figure 2N). The segmental expression persists longer in younger stages (Figures 2E-H and 3C) than in older stages (Figure 3D). Apart from its expression during segment formation, *Gm-run *is also expressed in broad, but well-defined, domains in the CNS (Figures 2G-L, N and 3D) including the ocular region (Figures 2F-H and 3C, D). This expression is initially weaker in the antennal and premandibular segment (Figures 2F-H and 3C). The dorsal expression in the postmaxillary segment is enhanced (Figures 2H and 3D).

**Figure 4:**
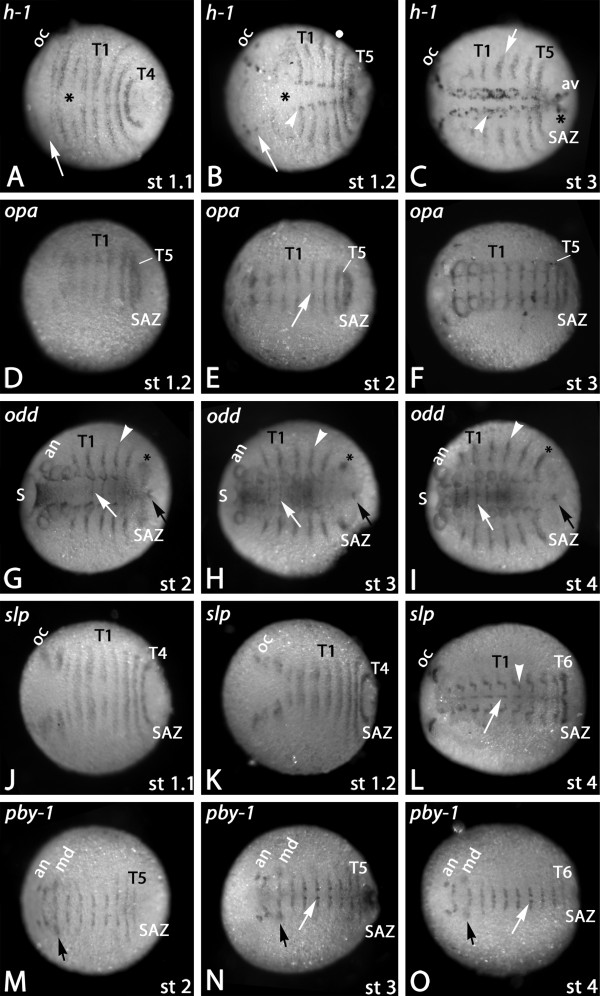
**Expression of *Glomeris hairy-1*, *odd-paired*, *odd-skipped*, *sloppy-paired *and *pairberry-1***. Expression of *hairy-1 *(*h1*) (**A-C**), *odd-paired *(*opa*) (**D-F**), *odd-skipped *(*odd*) (**G-I**), *sloppy-paired *(*slp*) (**J-L**) and *pairberry-1 *(*pby-1*) (**M-O**). (**A**) Stage 1.1 embryo. Arrow points to antennal segment that does not express *h1 *at this stage. Asterisk marks weaker expression near the ventral midline. (**B**) Stage 1.2 embryo. Asterisk as in (A). Note that the segmental expression proceeds into the extra-embryonic tissue (filled circle). Enhanced patch-like expression in the central nervous system (CNS) (arrowhead), the ocular region (oc) and the antennal segment (arrow). (**C) **Stage 3 embryo. Segmental stripes are restricted to dorsal tissue (arrow). Dotted expression in the CNS (arrowhead) and in the anal valves (asterisk). (**D) **Stage 1.2 embryo. (**E**) Stage 2 embryo. Expression is dis-continuous in the anterior segments (arrow). (**F**) Stage 3 embryo. Note that expression is restricted to ventral tissue only. (**G**) Stage 2 embryo. Asterisk marks dynamic expression in the SAZ. Arrowhead points to dorsal segmental stripes. White arrow points to ventral segmental stripes. Black arrow points to expression in the proctodaeum. (**H**) Stage 3 embryo. Note the transformation of the SAZ-expression (asterisk in (G)) into dorsal patches (asterisk in H) in parallel with a clearance of expression from the SAZ. Arrows and arrowhead as in (G). (**I**) Stage 4 embryo. Transformation of the dorsal patches (asterisk in (H)) into a dorsal stripe (asterisk in (I)). Arrows and arrowhead as in (G) and (H). (**J**) Stage 1.1 embryo. (**K**) Stage 1.2 embryo. (**L**) Stage 4 embryo. Segmental expression is reduced to the ventral midline (arrow), the ventral part of the appendages and small segmental domains in the CNS (arrowhead). (**M**) Stage 2 embryo. Note that expression does not extend into the appendage buds, except for the mandibles (black arrow). (**N**) Stage 3 embryo. Enhanced expression in the CNS (white arrow). Black arrow as in (M). (**O**) Stage 4 embryo. Arrows as in (N). Anterior is to the left in all pictures. Abbreviations as in Figure 2. Additional abbreviations: av, anal valves; S, stomodaeum

*Gm-h1 *is expressed in transversal segmental stripes at early segmentation stages (Figure 4A). Unlike *eve *and *run*, *h1 *is not expressed dynamically in the SAZ, but only appears at the very anterior of it, where it covers ventral and dorsal tissue (Figure 4A-C). Again the segmental expression is more obvious at younger stages. In older embryos the ventral segmental expression disappears soon after its appearance and only the dorsal segmental expression persists (Figures 4A-C and Additional file 1: Figure S1A, B). The dorsal stripes of *h1 *expression lie anterior to *en *(Additional file 1: Figure S1A, B). The dorsal expression reaches into the dorsal extraembryonic tissue (Additional file 1: Figure S1K). Apart from the expression during segment formation, *h1 *is also expressed in the anal valves (Figure 4C) and in part of the CNS including the ocular region (Figures 4B, C and Additional file 1: Figure S1C). The expression profile of *Gm-h2 *gene is similar to that of *Gm-h1 *(Additional file 1: Figure S1D/E). However, at younger developmental stages the expression is very weak compared to that of *h1 *and no dynamic expression in the SAZ is detectable. Like *h1*, also *h2 *is expressed in the dorsal embryonic tissue (Additional file 1: Figure S1L). *Gm-h3 *is expressed in the neuroectoderm, in segmental spots in the dorsal tissue, in the anal valves and at later stages in the tips of the appendages (Additional file 1: Figure S1F-H).

### Expression of secondary PRG orthologs during Glomeris trunk segmentation

The *Glomeris odd-paired *ortholog (*Gm-opa*) is expressed in segmental stripes. Its expression, however, is strictly restricted to the ventral part of the embryo (Figures 3E, F and 4D-F). *opa *is expressed posterior adjacent to the expression of *en *(Figure 3E, F). *opa *is also expressed in the SAZ (Figures 3E, F and 4D-F), but in a way that is different from the dynamic expression of *eve *and *run*. Initially the anterior SAZ expresses *opa *in a broad domain, that later transforms into a single small stripe in the newly formed segment (Figure 4D-F). *Gm-opa *is expressed in rings around the outgrowing appendages, with the exception of the labrum. The pre-mandibular and postmaxillary segments do not form appendages and here *opa *is expressed in the tissue corresponding to where the appendages form in the other segments (Figure 3E, F).

*Gm-odd *is expressed dynamically in the SAZ (Figure 4G-I). This dynamic expression, however, never results in ventral segmental stripes, but only in dorsal stripes. Initially a faint propeller-shaped expression domain appears in the SAZ (Figure 4G, I) that later becomes more clearly visible (Figure 4G). This domain then disappears from its ventral part and continues to strengthen in its dorsal part, leaving a strong patch of expression in the dorsal tissue of the nascent segment (Figure 4H). This then transforms into dorsal stripes (Figure 4I). The dorsal segmental expression of *odd *lies posterior to that of the segmental marker *engrailed *(*en*) (Additional file 1: Figure S1I, J). As with *h1 *and *h2*, *odd *expression also extends dorsally into the dorsal extraembryonic tissue (Additional file 1: Figure S1M). *Gm-odd *is also strongly expressed in the region of the forming stomodaeum (Figure 4G), the ventral portion of the developing appendages (Figure 4G), a dot-like domain anterior to the proctodaeum (Figure 4G-I) and in the CNS (Figure 4G-I).

*Gm-slp *is expressed in segmental stripes anteriorly abutting the expression of *en *(Figures 3G, H and 4J-L). The segmental expression initially extends into dorsal tissue, but soon after this expression pattern disappears (Figures 3G, H and 4L). There is no dynamic expression in the SAZ. The most posterior segmental stripe appears anterior to *en *in the anterior part of the SAZ or the nascent segment (Figures 3G, H and 4J-L). At later stages the segmental expression ceases in the anterior segments and remains only in the ocular region, the antennae, the walking legs, a restricted ventral region in the CNS, and segmentally reiterated stripes along the ventral midline (Figure 4L). The posterior part of the SAZ remains free from *slp *transcripts at all stages (Figures 3G, H and 4J-L).

*Gm-pby1 *is neither expressed dynamically in the SAZ nor in stripes in the posterior part of the SAZ, but appears first in a segmental stripe in the anterior of the SAZ or the nascent segment (Figures 3I, J and 4M-O). However, its expression is restricted to the ventral tissue only. The segmental expression disappears soon after its appearance in the ventral midline and the legs (Figures 3I, J and 4M-O). Its intra-segmental position overlaps with the posterior part of *wingless *(*wg*) (not shown) and the anterior part of *en *(Figure 3I, J). *Gm-pby2 *expression is restricted to the tips of the legs at late embryonic stages (not shown).

## Discussion

### Pair rule gene orthologs are involved in myriapod segmentation

**Figure 5:**
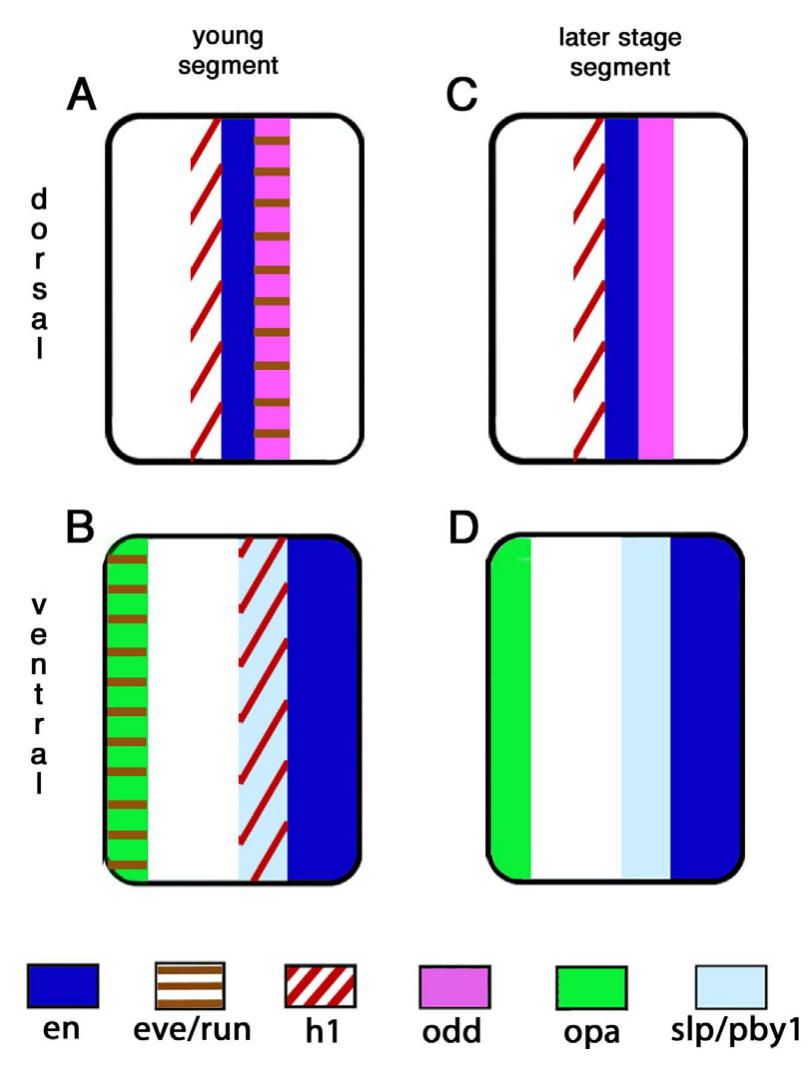
**Schematic summary of *Glomeris *pair rule gene expression patterns in dorsal and ventral segments**. Segmental expression of PRG orthologs in young **(A, B) **and older **(C, D) **segments. (B) and (D) represent ventral segments, (A) and (C) represent dorsal segments. The genes are denoted in different colors and patterns and are explained in the legend below the drawings. Note that expression of the primary PRGs *eve *and *run *is present in the nascent segments, but absent from older segments, both in ventral and dorsal tissue. The patterning of dorsal and ventral segments is very different at the level of the PRGs. Some genes are exclusively expressed in dorsal or ventral segments, respectively (for example, *odd*, *pby1*, *opa*). Other genes are expressed in both ventral and dorsal segments, but show differences in their temporal expression profile or their intrasegmental position (for example, *eve*, *run*, *h1*). These results support earlier results that suggested a decoupling between dorsal and ventral segments, and indicate that this decoupling is already present at the level of the PRGs. Abbreviations: *en*, *engrailed*; *eve*, *even-skipped*; *h1*, *hairy1*; *odd*, *odd-skipped*; *pby1*, *pairberry1*; *run*, *runt*; *slp*, *sloppy-paired*; *wg*, *wingless*.

So far only expression data of a few PRG orthologs in centipede myriapods (Chilopoda) have been published, but no data have been available for PRG orthologs of millipedes (Diplopoda). Since functional studies have not yet been established for any myriapod species our knowledge on PRGs in myriapods is restricted to sequence and expression data [16-18]. All canonical PRGs are transcription factors with a spatially and temporally regulated expression pattern and it is reasonable to suspect that they should, therefore, be functional more or less exclusively in those cells where they are expressed. Thus, in the absence of functional data the PRGs expression data can provide us with clues as to their likely role in segmentation. Segmentation genes on the level of the segment polarity genes (SPGs) and PRGs are in most cases expressed in transversal stripes within the segments or at least in the posterior SAZ where the segments are formed, clearly suggesting a role in segment specification and formation (Figure 5). Some functional studies in insects, however, report possible exceptions from this rule. In *Tribolium*, for example, RNAi did not uncover a function of the primary PRG *hairy *in posterior segmentation [31], although its expression pattern suggests this [32,33]. Furthermore, in the same species *fushi-tarazu *(*ftz*) and *odd-paired *(*opa*) did not show segmentation defects in RNAi studies, although they are expressed in the typical transversal stripes [9,34]. In most cases, however, gene expression data in comparable developmental studies give a valuable clue on what a gene's function may be and if this function could be conserved in different organisms.

The expression profiles of the PRGs examined here suggest a general role in segment formation, or at least the development of segmentally-iterated structures. Even if some of the reported PRGs in *Glomeris *- despite their expression patterns that are typical for segmentation genes - might not be involved in segmentation, the data from a wide range of arthropods including spiders and myriapods support the idea that PRGs are generally important factors in arthropod segmentation [13,21].

### Posterior segments form in a single segmental periodicity

It is still an open question whether PRGs act in a pair rule like manner in arthropods other than *Drosophila *and *Tribolium *[1,9,10,35].

In the hemimetabolous insect *Schistocerca americana *and the spider-mite *Tetranychus urticae *the *prd/pby *orthologs *Sg-pby1 *and *Tu-Pax3/7 *appear with delay in every other segment in the anterior embryo which may hint at an ancestral double segmental mechanism [36,37]; discussed in [2]. However, outside the higher insects such a pair rule mechanism may be restricted to the anterior part of the embryo, which is patterned similarly as the *Drosophila *embryo. Therefore, mechanisms found in anterior patterning are likely to be more conserved between short germ and long germ arthropods than it is the case for posterior patterning (discussed in [38-40]). Since expression data on PRGs in anterior patterning are scarce outside the insects, this theory remains open for further debate and study.

There is, however, one example that supports a posterior pair-rule like mechanism outside higher insects. In the centipede *Strigamia maritima *posterior segments are added with a double segmental periodicity [17,18]. This pair-rule like mechanism appears, however, to be the result of parallel evolution rather than a conserved feature of arthropod posterior segmentation, because some of the genes involved, like, for example, *caudal*, appear to be co-opted for this special purpose [17], discussed in [41,42]. The expression of another PRG, *eve*, in the centipede *Lithobius atkinsoni*, however, does not show any kind of a double segmental pattern [16] further supporting the thesis that the double segmental mechanism found in *Strigamia *may be a peculiarity of geophilomorphs rather than a general feature of myriapods or even short germ arthropods. This is also supported by data from the spider *Cupiennius salei *where PRGs show no double segmental expression [13,21,22].

We find that none of the investigated PRGs are expressed in alternating segments in the trunk. In fact, the dynamic expression patterns of *Gm-eve *and *Gm-run *demonstrate clearly that the posterior trunk segments appear with a single segmental period (Figure 2). The dynamic expression in the SAZ of either of these genes can be followed into each nascent segment demonstrating that every single stripe of *eve *or *run *is correlated with the formation of a single new posterior segment.

### Conserved aspects of early PRG interaction

In *Drosophila *the PRGs are subdivided into two classes; the primary PRGs, which are under control of maternal factors and the gap genes; and the secondary PRGs, which receive their input from the primary PRGs [1]. Recent studies in the beetle *Tribolium *have examined the possibility of this PRG hierarchy being conserved [9]. Although a number of differences exist in the regulation of the PRGs, the principal hierarchy of PRGs between *Drosophila *and *Tribolium *is conserved. *eve *and *run *act high in the regulatory network in both species [9,43-45] and *prd *and *slp *are under the control of these primary PRGs [9,45,46]. Data from the spider *Cupiennius *support the idea that a hierarchic order of PRGs may even be conserved in all arthropods, since *Cs-eve *and *Cs-run *are expressed more posterior (that is, earlier) in the SAZ than *Cs-slp *and *Cs-pby-3 *that are restricted to the anterior of the SAZ and the anterior rim of the SAZ, respectively [13,21,22].

In *Glomeris eve *and *run *both display the most prominent expression pattern in the SAZ passing through it from its very posterior to its anterior and finally into the nascent segments (Figure 2). Furthermore these two PRG orthologs are the only ones in *Glomeris *being additionally expressed in concentric circles around the proctodaeum, as is the case for *odr-1 *in *Strigamia *[17] or *eve *in *Lithobius *[16] (Figure 2M, N). The dominant expression profiles of *eve *and *run *in the SAZ thus make them good candidates for segmentation genes acting at high level in a possible network, as it is the case for their orthologs in *Drosophila *[45], *Tribolium *[9] and possibly also *Cupiennius *[13,21].

The *Glomeris *orthologs of the secondary PRGs (that is, *Gm-slp *and *Gm-pby1*) are never expressed dynamically in the SAZ, but appear relatively late in the SAZ shortly before the inter-segmental borders form (Figure 4J-O). Notably these two genes also display highly conserved intra-segmental expression patterns (discussed below). In addition, both orthologs are never expressed in the posterior part of the SAZ in *Cupiennius*, but only in the anterior SAZ (that is, *Cs-pby-3*) or at the anterior rim of the SAZ (that is, *Cs-slp*) [13]. This led Damen and colleagues [13] to suggest that these genes may be under the control of genes like *eve *and *run*, which are active earlier (that is, already in the posterior SAZ). Since the relative tempo-spatial expression of some of the putative primary and secondary PRGs in the SAZ is conserved between *Tribolium*, *Cupiennius *and *Glomeris*, we postulate that at least *eve *and *run *are acting at a high level in a possible regulatory network of PRG orthologs in all arthropods.

In *Glomeris *the *odd *gene, which is a primary PRG in *Tribolium *and a secondary PRG in *Drosophila*, is also expressed dynamically in the entire SAZ (Figure 4G-I), suggesting that *odd *may play an important role in segment formation in *Glomeris*. Moreover, expression of the *odd *ortholog *odr-1 *in the centipede *Strigamia *also suggests a crucial role of this gene in posterior segment formation [17]. Taken together these data imply that *odd *may already have gained a dominant function in segmentation in lower arthropods. Later, it may then have been recruited as a primary PRG in *Tribolium*. Indeed, in a bioinformatics study on transcriptional regulation of segmentation gene interaction in *Drosophila odd *rather behaves like a primary PRG although functionally it is a secondary one [47]. This result strengthens the idea that *odd *orthologs fulfill important tasks in arthropod segmentation, even in *Drosophila*, where its function in segmentation may be understated by the fact that it acts like a secondary PRG.

### Conserved segmental expression of secondary PRGs

As already discussed above, there appears to be a hierarchy of PRGs in *Glomeris *where putative primary PRGs are expressed early and dynamically in the *Glomeris *SAZ; whereas the expression of putative target genes such as the secondary PRGs (and SPGs) is restricted to anterior regions of the SAZ and the nascent segment. However, in *Drosophila *some of the PRGs are also expressed at later stages, where they function to stabilize the parasegment boundary (PSB) [4]. Several studies have shown that the parasegment boundary is a conserved entity among arthropods and characterized by the expression of SPGs, like *engrailed *and *wingless*, at either side of the parasegment boundary [8,19,29,48-51].

We, therefore, also analyzed the intra-segmental expression of the *Glomeris *PRGs and discuss here to what extend this late function/interaction of the *Glomeris *PRGs may be conserved or have diverged. Expression and function of most PRGs is known only from *Drosophila *and *Tribolium*, which are both rather derived holometabolous insects. Apart from that, the only almost-complete set of PRG orthologs is known from the spider *Cupiennius*. But the focus of this study is on the early expression in the SAZ and does not include the examination of later segmental expression patterns [13]. The data on "late" PRG expression are thus very limited except for data on *Pax3/7 *group genes (that is, the *Gm-pby1 *orthologs) that are very abundant due to the availability of cross-reacting antibodies detecting all genes of this family in various arthropods (that is, orthologs of *Drosophila paired, gooseberry and gooseberry-neuro*) [15,36].

The segmental expression patterns of the primary PRGs in *Glomeris *are different from that in *Tribolium *and/or *Drosophila*. For example, *Gm-eve *and *Gm-run *are expressed posterior to *Gm-en *in the ventral segments with no or only very little overlap (Figure 3A-D), whereas, in *Drosophila *and *Tribolium eve *and *run *expression is overlapping with *en *[9].

**Figure 6:**
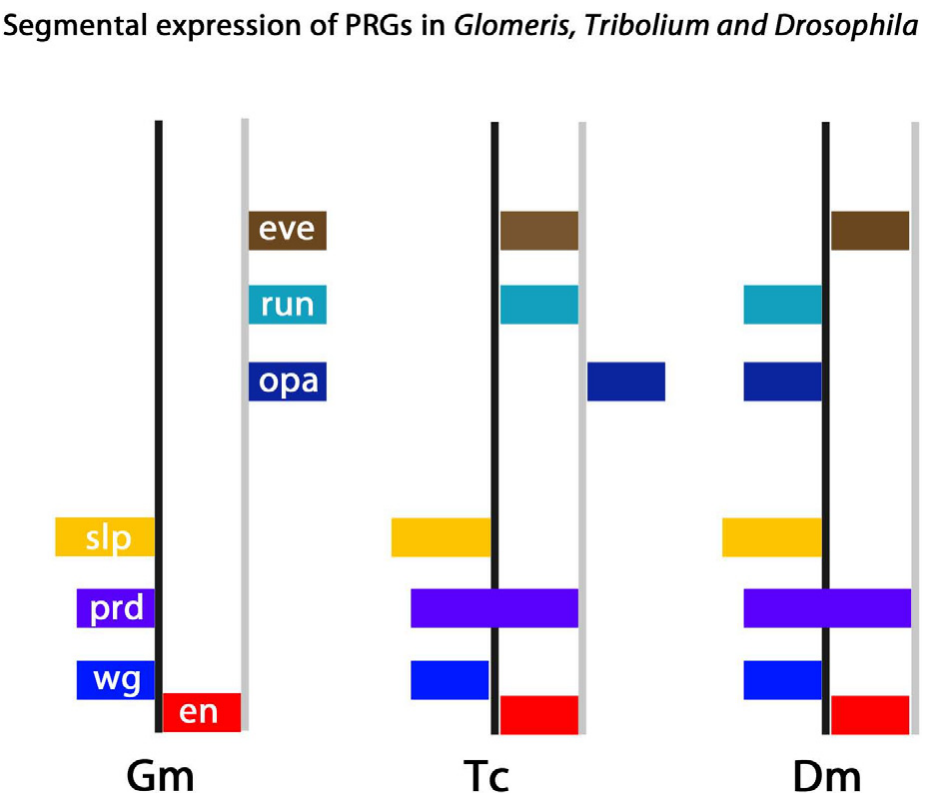
**Segmental expression of PRGs in *Glomeris (ventral segments only), Tribolium and Drosophila***. Schematic drawing and colour coding is based on [70]. Expression of *en *(red), *wg *(blue), *prd *(purple), *slp *(yellow), *opa *(dark blue), *run *(petrol) and *eve *(brown). Black lines represent parasegment boundaries; grey lines represent segment boundaries. The expression of the segment polarity genes *en *and *wg *is conserved, as well as that of the PRG *slp*. Expression of *prd *is conserved with modification in every other segment in *Tribolium *and *Drosophila*. Intra-segmental position of *opa *is conserved between *Tribolium *and *Glomeris*, which may reflect the ancestral pattern. Co-expression of *eve *and *run *is also conserved *Tribolium *and *Glomeris*, but at a different intra-segmental position.

The segmental expression of the secondary PRG *opa *is only reported from the fly *Drosophila *and the spider *Cupiennius*. In the fly an initial ubiquitous expression later refines into faint segmental stripes, but the intra-segmental position of these stripes is unclear [52]. In the spider the gene is expressed in stripes in the SAZ and the newly formed segments, but again the intra-segmental position of its expression is unclear [13]. In *Tribolium *however *opa *is expressed posterior abutting to *en *(with little overlap) (CP Choe and SJ Brown personal communication). This expression is thus conserved at least between *Tribolium *and *Glomeris*, and may therefore represent the ancestral expression profile of *opa *(Figure 6).

The intra-segmental expression of *Gm-slp *and *Gm-pby1 *is conserved between *Glomeris *and various arthropods (for *pby1*) [15,36] and *Glomeris*, *Drosophila *and *Tribolium *(for *slp*) [5,10]. In all cases *slp *is expressed anteriorly adjacent to *en*, and the *Pax3/7 *ortholog *pby-1 *is expressed overlying the PSG boundary and, thus, partially overlapping the expression of *wg *and of *en *(Figure 6).

We, therefore, propose that the late function of *slp *and *Pax3/7 *orthologs is conserved in the formation of the PSB in that *slp *represses *en*, and in that *pby1 *might activate *wg *and *en*. Whether the segmental expression profiles of the other *Glomeris *PRG orthologs is conserved among basal arthropods and what their late function in segmentation may be has to be examined in the future. Data on expression and function of all PRG orthologs from various basal arthropods would possibly help in answering this question.

### Decoupled dorsal and ventral segmentation

In *Glomeris *the germ band consists of ventral tissue from the beginning of germ band extension on, but dorsal tissue does not start developing before stage 3 (staging after [19]). We reported earlier that *Glomeris *dorso-ventral segmentation is decoupled at the level of the segment polarity (SPGs) and Hox genes suggesting that the process of decoupling must have its origin at earlier regulatory levels [19,20]. Since in *Drosophila*, the SPGs are under control of the pair rule genes (PRGs) it appeared likely that the decoupling of segmentation in *Glomeris *is attributed to the level of the PRGs. Our screen on *Glomeris *PRGs now revealed that some genes display a profile that suggests restricted function in dorsal segmentation, while others are predominantly expressed in ventral tissue (Figure 5). By contrast, the genes *opa *and *pby-1 *are expressed in stripes only in the ventral segments. Thus, they are likely to be involved in ventral segmentation, but they are not required for the formation of the dorsal segments. The remaining genes are initially expressed in ventral and dorsal segmental tissue, but show dorso-ventral differences in their persistence of expression. The expression of *eve*, *runt *and *h1 *stripes persist longer in dorsal tissue, whereas the *slp *stripes persist longer on the ventral side (Figures 4 and 5). Our data thus suggest that decoupling of dorsal and ventral segmentation already starts with the action of the PRG orthologs. Especially those genes that are initially expressed on both sides, but then "decide" for either ventral or dorsal persistence, provide further insight into the possible mechanisms of dorso-ventral decoupling. These genes are likely members of a core segmentation network that is common to both ventral and dorsal segments, but then are regulated differently on both sides to achieve the specific metamerisms of the ventral and dorsal sides, respectively.

The finding that the ventral segmentation gene expressions in *Glomeris *show similarities to those in other arthropods not only on the level of the SPGs but also of the PRGs, while the dorsal patterns deviate on this level as well, strengthens the notion that the ventral metamerism in *Glomeris *is homologous to the segmentation in other arthropods, while the dorsal metamerism mechanisms have diversified independently in different arthropod lineages [19].

### The idea of an arthropod "segmentation clock"

Segmentation in short-germ arthropods and vertebrates displays fundamental morphological similarities. Separate body units (that is, segments and somites, respectively) are added sequentially from a posterior region in the embryo: the SAZ in arthropods and the presomitic mesoderm (PSM) in vertebrates [53,54]. The cyclic expression of genes that is involved in the formation of new somites in vertebrates very closely resembles the dynamic expression of the *Glomeris *PRGs *eve*, *run *and also *odd*. The cyclic gene expression in vertebrates is under control of a so-called "segmentation clock" or "oscillator", of which Notch/Delta signaling is a key component [54]. Recent work on the spider *Cupiennius salei *and the cockroach *Periplaneta americana *[55,56] has shown that Notch/Delta signaling is a main component of arthropod segmentation as well, and implies that the lack of Notch/Delta signaling in *Drosophila *segmentation is the derived state. The morphological similarities of segment formation in arthropods and vertebrates and the common usage of Notch/Delta expression suggest that segmentation in both phyla traces from a common ancestor [55]. However, the functional similarities between segmentation in arthropods and vertebrates are more extensive than just using Notch/Delta signaling. Recent work in the spider *Achaearanea tepidariorum *has shown that a posterior expression of the *Wnt8 *gene is involved in the maintenance of SAZ activity [57-59] and the ortholog of this gene, *Wnt8 *is required for the proper function of the PSM in vertebrates [60,61].

It is currently unclear how these newly discovered vertebrate-like mechanisms feed into the more canonical segmentation mechanisms in arthropods, that is, the level of the pair rule and segment polarity genes. It has been suggested that these levels are under control of a posterior segmentation clock involving Notch/Delta signaling [39]. So far there is only circumstantial evidence that supports this idea. First, the vertebrate *Her/Hes *genes, that are orthologs of the arthropod PRG *hairy*, are controlled by the Notch/Delta oscillator [62,63], thus providing a link between these two components in vertebrates. It is possible that this link has been conserved and extended during arthropod evolution. Indeed, in the spider *Cupiennius hairy *expression is changed upon *Notch *and *Delta *RNAi [55,64]. Second, the PRG *odd *is regulated downstream of Notch signalling in *Drosophila *leg development [65]. This fact caused Chipman *et al. *[17] to suggest that *odd *might be under the control of Notch/Delta signaling in other processes as well, including segment formation. In agreement with this idea, in *Glomeris *and two other myriapods, *Strigamia *and *Lithobius*, *Notch *and *Delta *are expressed dynamically in the SAZ [18,66,67], strikingly similar to the dynamic patterns that we find in the present work for the segmentation genes. In *Glomeris *this dynamic *Notch *pattern results in dorsal segmental *Notch *stripes [66] and we have shown here that the *odd *stripes in *Glomeris *also persist only in the dorsal segments, thus pointing to a possible link between Notch signaling and *odd *activation. Another argument for the involvement of Notch/Delta signalling in dorsal segment formation comes from the expression of *Gm-h1 *(Figure 4B, C). Like *Gm-Notch *and *Gm-odd *also *Gm-h1 *becomes restricted to dorsal segments soon after its expression in the SAZ (Figure 4B, C). The expression profiles of *Notch*, *Delta*, *odd*, and *h1 *would be in agreement with the suggested ancestral interaction of a posterior "segmentation clock" mechanism and PRG orthologs [39,68].

### Expression of PRGs in dorsal extraembryonic tissue

In most arthropods a thin layer of dorsal ectodermal tissue connects the separated dorsal edges of the developing embryo [69]. The nature and function of this tissue is not very well understood and the lack of expression of the segmentation genes in this tissue in most species suggests that it is not metamerized or subdivided into separate developmental units. However, in *Glomeris *the primary PRGs, *eve *and *run *are expressed in circles around (or within) the SAZ, which is especially clear at early developmental stages (Figure 2); the segmental expression of these genes thus includes extraembryonic tissue. A similar expression profile has also been reported for pair rule genes, for example, *odr1 *(*odd *ortholog) and *eve1/2*, in *Strigamia *[18] and it was, thus, not unexpected to find similar patterns in *Glomeris *as well. In *Glomeris*, however, *h1 *and *odd *are not only expressed in the dorsal segments (discussed above), but their segmental expression in the extraembryonic tissue persists even in older stages (Additional file 1: Figure S1). Since neither the segment polarity genes nor the Hox genes are expressed in the extraembryonic tissue it appears unlikely that this tissue is patterned or metamerized in the classical sense. But what then is the function of *h1*, *h2 *and *odd *expression in the extraembryonic tissue?

We postulate that the expression of the *hairy *genes (*h1 *and *h2*) and *odd *may be involved in "guiding" the two dorsal sides of the embryo in order to assure that "matching" tissue meets during the process of dorsal closure. This would be especially important, because the matching cannot be guided via the connection across the ventral side, as the dorsal segments are functionally decoupled from the ventral side.

## Conclusions

The activation of the pair rule genes (PRGs) appears to be a crucial step in arthropod segmentation as it marks the transition from a non-periodic pattern to a periodic pattern. It is, therefore, surprising to some degree that this step of the segmentation mechanisms appears to be the most variable and diverse level, especially when it is compared to the evolutionarily conserved steps of the SPGs or the Hox genes [40,70,71]. Expression of all investigated PRGs in the millipede *Glomeris *is consistent with a role in segmentation. The PRGs in *Glomeris *seem to act in a hierarchic manner, and can be subdivided into primary and secondary PRG, similar as has been described for insects and a spider [9,13]. However, the exact interactions between the *Glomeris *PRGs remain unclear due to the lack of functional methods. The expression of the primary PRGs strongly supports the idea that the initial patterning of the segments is in a single segmental period. A similar single segmental period has also been observed in other arthropods implying that it represents the ancestral mode for segment patterning and formation [22].

Our work is the first analysis in a non-insect arthropod in which the late expression of the PRGs has been studied. The intrasegmental positions of the expression patterns show similarities but also differences among the species analysed (Figure 6A). The comparison of insect data (that is, *Tribolium *and *Drosophila*) with data from a myriapod may help to distinguish between conserved and derived features of segmentation and thus provide clues about the origin and evolution of segmentation mechanisms in the arthropods.

In both *Tribolium *and *Glomeris *the intra-segmental expression of *opa, slp *and *prd/pby1 *is conserved, and although its function is unclear, it may be part of the ancestral patterning system (Figure 6).

An interesting case of "partial" conservation is provided by the genes *run *and *eve*. These genes are co-expressed in *Tribolium *and *Glomeris *(but not in *Drosophila*) (Figure 6). However, the co-expression stripes of *run *and *eve *do not have the same intrasegmental position in the two species. Thus, co-expression of these two genes appears to be a conserved feature already present in the common ancestor of insects and myriapods, but the mechanisms that position the *run/eve *stripe within a segment must differ in the two species.

Our results also add to the previous finding that the segmentation mechanisms in the dorsal and ventral segments of *Glomeris *are decoupled [19]. Previous results have shown that dorsal and ventral segmentation mechanisms differ mainly in their usage of the segmentation genes of the segment polarity group, but our present data show that strong dorso-ventral differences already exist in the expression of the orthologs of the PRGs.

The ancestral role of the PRGs in arthropod segmentation remains unclear until comparative data on expression and function are available for representatives of all groups of arthropods, as well as their assumed sister-groups, the onychophorans and the tardigrades [72]. Shared conserved or diverged aspects of the segmentation process may then also contribute to the unraveling of arthropod phylogeny [73], which appears still not fully resolved [74,75].

## Competing interests

The authors declare that they have no competing interests.

## Authors' contributions

RJ carried out the experiments, wrote the first draft of the manuscript and was mainly responsible for the experimental outline. GEB, NMP and WGMD were involved in drafting the final version of the manuscript. NMP and WGMD discussed the experimental outline. RJ and WGMD initiated work on *Glomeris *segmentation.

## Supplementary Material

Additional file 1 **Figure S1. Additional aspects of *h1 *and *odd *expression, expression of *h2 *and *h3 *and single-colour expression detection of *h1+en *and *odd+en*. (A, B) **Double single-colour staining of *h1+en*. The black arrows point to expression of *en*; the white arrows point to expression of *h1 *anteriorly abutting the expression of *en*. (**C) **Expression of *h1 *in a stage 5 embryo (cf. similar expression of *h2 *in (E)). White arrow points to expression in the ventral nervous system. Asterisk marks expression in the brain. (**D, E) **Expression of *h2*. White arrow and asterisk in (E) as in (C). (**F-H) **Expression of *h3*. Arrows in (F) point to expression in the brain and the ventral nervous system. Arrowhead points to expression in the SAZ. Asterisk marks expression in the labrum. (G) Same embryo as in (F) but slightly rotated to show a rear aspect and the posterior expression in the SAZ (arrowhead). (H) De novo expression in lateral tissue (arrow) and in the appendages (arrowhead). (H') Same embryo as in (H); DAPI stained; arrow and arrowhead point to same position as in (H). (**I) **Double single-colour staining of *odd+en*. (**I') **Same embryo as in (I); DAPI stained. (**J) **Close-up on dorsal tissue of the embryo shown in (I/I'). Black arrow points to expression of *en*. White arrow points to expression of *odd*. (**K) **Dorsolateral view of a stage 5 embryo stained for *h1*. Dorsal segmental expression is connected by weak expression (asterisks) in the dorsal extraembryonic tissue (e). (**L) **Dorsal view. Expression of *h2 *in the (e). Asterisks as in (K). (**M) **Dorsal view. Expression of *odd *in the (e). Asterisks as in (K). Abbreviations: (e), dorsal extraembryonic tissue; *en*, *engrailed*, *h*, *hairy*; *odd*, *odd-skipped*; st, stage. Click here for file
