# Supplementary figures and images for: Expression of myriapod pair rule gene orthologs
Source: EvoDevo. 2011 Feb 25;2:5. doi: 10.1186/2041-9139-2-5 (PMC3058060; doi:10.1186/2041-9139-2-5)

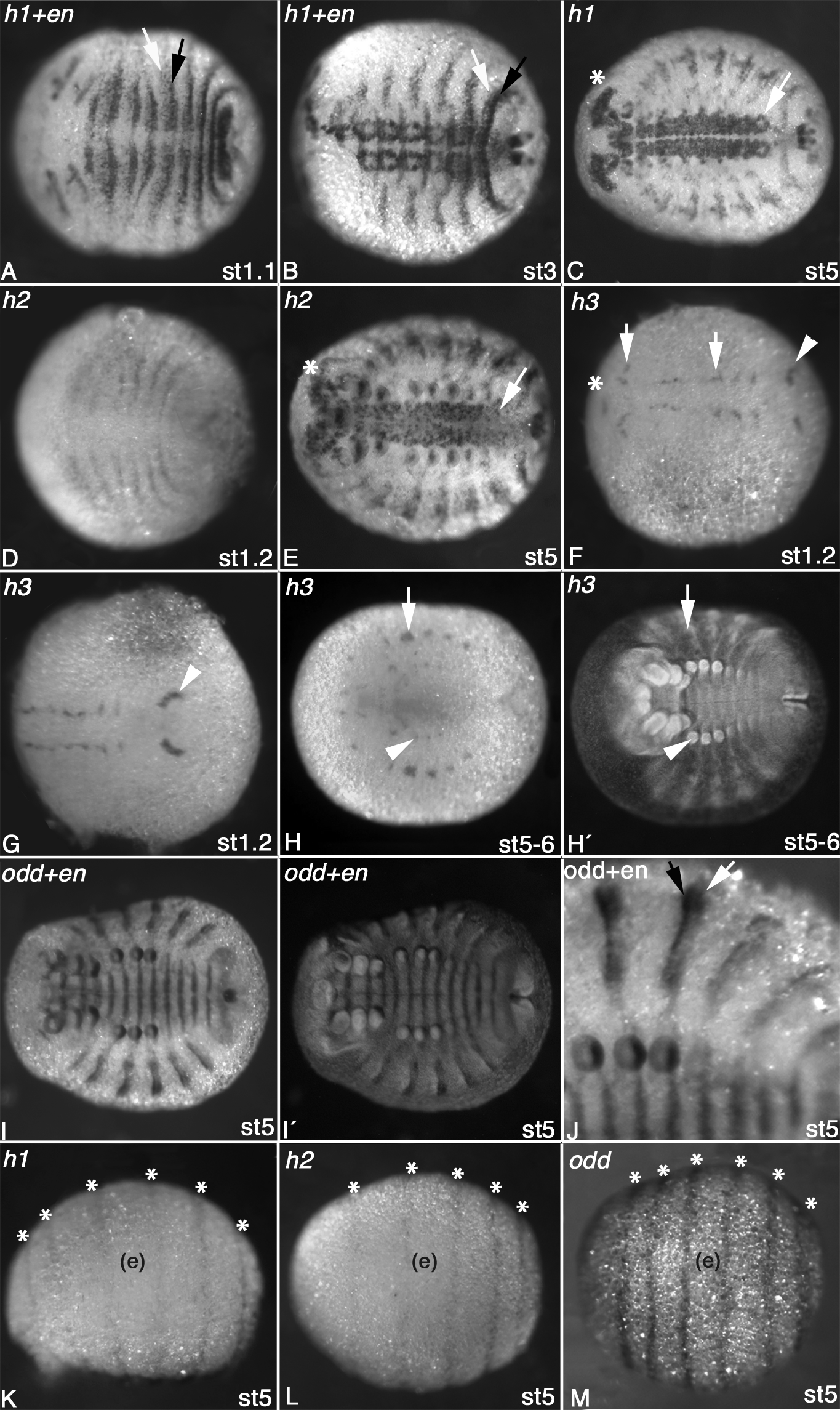

Supplement: Additional file 1 — Figure S1. Additional aspects of h1 and odd expression, expression of h2 and h3 and single-colour expression detection of h1+en and odd+en. (A, B) Double single-colour staining of h1+en. The black arrows point to expression of en; the white arrows point to expression of h1 anteriorly abutting the expression of en. (C) Expression of h1 in a stage 5 embryo (cf. similar expression of h2 in (E)). White arrow points to expression in the ventral nervous system. Asterisk marks expression in the brain. (D, E) Expression of h2. White arrow and asterisk in (E) as in (C). (F-H) Expression of h3. Arrows in (F) point to expression in the brain and the ventral nervous system. Arrowhead points to expression in the SAZ. Asterisk marks expression in the labrum. (G) Same embryo as in (F) but slightly rotated to show a rear aspect and the posterior expression in the SAZ (arrowhead). (H) De novo expression in lateral tissue (arrow) and in the appendages (arrowhead). (H') Same embryo as in (H); DAPI stained; arrow and arrowhead point to same position as in (H). (I) Double single-colour staining of odd+en. (I') Same embryo as in (I); DAPI stained. (J) Close-up on dorsal tissue of the embryo shown in (I/I'). Black arrow points to expression of en. White arrow points to expression of odd. (K) Dorsolateral view of a stage 5 embryo stained for h1. Dorsal segmental expression is connected by weak expression (asterisks) in the dorsal extraembryonic tissue (e). (L) Dorsal view. Expression of h2 in the (e). Asterisks as in (K). (M) Dorsal view. Expression of odd in the (e). Asterisks as in (K). Abbreviations: (e), dorsal extraembryonic tissue; en, engrailed, h, hairy; odd, odd-skipped; st, stage. [file 2041-9139-2-5-S1.TIFF]
